# Editorial: Integrating Computational and Neural Findings in Visual Object Perception

**DOI:** 10.3389/fncom.2016.00036

**Published:** 2016-04-20

**Authors:** Judith C. Peters, Hans P. Op de Beeck, Rainer Goebel

**Affiliations:** ^1^Cognitive Neuroscience Department, Faculty of Psychology and Neuroscience, Maastricht UniversityMaastricht, Netherlands; ^2^Neuroimaging and Neuromodeling Department, Netherlands Institute for NeuroscienceAmsterdam, Netherlands; ^3^Laboratory of Biological Psychology, University of LeuvenLeuven, Belgium

**Keywords:** object recognition, computer vision, fMRI, feature representation, ventral visual pathway, invariance

Recognizing objects despite infinite variations in their appearance is a highly challenging computational task the visual system performs in a remarkably fast, accurate, and robust fashion. The complexity of the underlying mechanisms is reflected in the large proportion of cortical real-estate dedicated to visual processing, as well as in the difficulties encountered when trying to build models whose performance matches human proficiency.

The articles in this Research Topic provide an overview of recent advances in our understanding of the neural mechanisms underlying visual object perception, focusing on integrative approaches which encompass both computational and empirical work. Given the vast expanse of topics covered in the discipline of computational visual neuroscience, it is impossible to provide a comprehensive overview of the field's status-quo. Instead, the presented papers highlight interesting extensions to existing models and novel insights into computational principles and their neural underpinnings. Contributions could be coarsely subdivided into three different sections: Two papers focused on implementing biologically-valid learning rules and heuristics in well-established neural models of the visual pathway (i.e., “VisNet” and “HMAX”) to improve flexible object recognition. Three other studies investigated the role of sparseness, selectivity, and correlation in optimizing neural coding of object features. Finally, another set of contributions focused on integrating computational vision models and human brain responses to gain more insights in the computational mechanisms underlying neural object representations.

## Extending invariant recognition capabilities of existing models

A key challenge our visual system faces is a trade-off between discrimination and generalization. It should be able to discriminate an encountered object from a myriad of possible alternatives. Yet, it has to generalize across different instances of the same object, or, in other words, be invariant to so-called “identity-preserving transformations” (DiCarlo et al., 2012). Two contributions in this Research Topic propose updates to influential computational models to more adequately deal with the latter invariance constraint.

Rolls and Webb, introduce an extension of the Ventral Visual Stream (VVS) model “VisNet” (Rolls, 2012) by incorporating a bottom-up driven saliency-detection mechanism to locate items of interest in natural scenes. By adding this functionality, their model mimics the “divide-and-conquer” strategy applied by the primate visual system: the dorsal stream uses stimulus saliency to guide saccades, which then allows the VVS to successively process a set of relatively small fixated regions (instead of having to deal with a complex visual scene in its entirety), thereby reducing the computational requirements to achieve invariant object recognition. The presented results show that VisNet could reliably locate and identify a number of objects in cluttered scenes, portraying both view and translation invariance, even though training encompassed only four viewpoints and a limited range of positions per object. These findings further corroborate the notion that learning rules based on temporal continuity (i.e., exploiting the increased likelihood that consecutive retinal images belong to the same object despite slight changes in its appearance) can successfully guide the development of invariant object representations.

Likewise, Parker and Serre show that another prominent model, namely HMAX (Riesenhuber and Poggio, 1999), can be extended to learn invariant recognition across 3D-rotations (while previous instantiations were limited to 2D changes in position and scale) based on unsupervised training on short object transformation sequences. The extended model exhibited greater sensitivity to so-called “Non-Accidental Properties” (akin to infero-temporal cortical responses) and concomitantly demonstrated greater tolerance to object transformations in its input.

## Efficient neural coding strategies

The selectivity and sparseness observed in neural firing elicited by visual stimulation are generally considered hallmarks of an efficient coding scheme: since a given neuron only responds to a limited set of inputs, and conversely any input only triggers activity in a relatively small fraction of the neural population, redundancy is minimized. In their contribution, Xiong et al. show that both selectivity and sparseness (which need not be correlated) can simultaneously arise as properties of modeled V1 receptive fields by reinforcing diversity (i.e., minimizing similarity by mimicking neural inhibition) during the training of a restricted Boltzmann machine (a type of network routinely used in “deep learning” approaches LeCun et al., 2015).

Interestingly, the findings presented by Hung et al. actually point to a role of *correlated* neural activity in efficient visual recognition as opposed to the proposedly beneficial de-correlation that tuning selectivity might offer. Based on dense neurophysiological recordings in monkey infero-temporal cortex, the authors show that correlation strength and tuning selectivity are only weakly related and that the observed correlated activity is mainly driven by neurons in IT output layers that convey generalizable object information, which is behaviorally relevant as it predicts human visual search performance (see below). Relatedly, Gladilin and Eils discuss the behavioral and neural importance of (phase) correlation in visual input.

## Linking computational models to human brain responses

Human neuropsychological and neuroimaging studies have consistently identified brain regions involved in object recognition. Nevertheless, our current understanding of ongoing computations and feature representations within these areas is rather limited.

One way forward to unravel the identified regions' inner workings is to compare the similarity across neural response patterns elicited by a given stimulus set to the similarity in output of a range of computer-vision models (with different feature extractions) when presented with the same stimulus set. Using this exploratory strategy, Aminoff et al. demonstrate that fMRI activation-patterns within scene-selective brain regions, such as the parahippocampal (PPA) and occipital place area (OPA), correlated most strongly with computer-vision models incorporating semantic features. In comparison, correlations were lower for models representing low-level features and for behavioral similarity scores. Conversely, the activation-pattern observed in the retrosplenial complex (RSC) was more in line with one of the low-level models and did correlate with subjective similarity ratings. Although encouraging, the results also clearly indicated that the overall correspondence between empirical and modeled responses was weak, suggesting that we still lack a clear grasp on cortical feature representation. One such feature, visual texture, is further explored in the contribution by Liu et al. using behavioral methods and modeling.

Another approach to gain insights into VVS feature representations is employed by Lescroart et al. They compared how well three encoding models, based on different scene-defining feature classes, could voxel-wise predict neural representations in scene-selective brain regions. The encoding models mapped a diverse set of natural images to three qualitatively different feature spaces: 2D-features related to Fourier-power, the subjective 3D—distance to salient objects in the scene, and a more abstract, semantic scene description (“object-categorization”). In line with Aminoff et al. the object-category model provided a better prediction of PPA and OPA activity compared to the other two encoding models which did not include semantic features. In addition, RSC activity was more accurately predicted by the object-category model than the Fourier-power model, but the object-category model and the 3D-distance model performed equally well. Although, results of both studies suggest a different feature representation for scenes in RSC compared to PPA and OPA, it should be noted that feature representations in all areas are more complex than captured by the applied computer-vision and encoding models. Response variance explained by the models was largely shared in the fMRI data of Lescroart et al. To which extent this reflects an actual combined representation of the model's different feature classes, or alternatively the high correlation between these feature spaces in natural images, could be further explored by follow-up studies using stimulus sets with reduced feature covariance (yet covering enough variance for real-world generalization). Furthermore, such studies might attempt to establish new encoding models based on feature spaces inspired by feature representations in high-level computer-vision models (e.g., Aminoff et al.) or deep neural nets (e.g., Güçlü and Van Gerven, 2015).

However, even the most optimal feature representations based on such approaches currently miss an important ingredient that might be essential for our fast and efficient object recognition: feature representations in the brain are dynamically influenced by task demands. We actively engage in a dynamical world, intentionally searching for and interacting with objects, rather than passively observing static sceneries. Several aforementioned contributions highlight specific aspects of such active perception, and more aspects can be distinguished. For example, to selectively process objects of interest over distracting information, we can use (c)overt spatial attention to constrain computations (see Rolls and Webb), but also non-spatial attention contributes to an efficient read-out of neural representations by altering the corresponding feature space. In particular, during visual search for objects in a movie, fronto-parietal and occipito-temporal activations become tuned toward the attended object-category, expanding representations of this and semantically related categories, at the cost of unattended categories (Çukur et al., 2013). Likewise, the work by Hung et al. revealed that proximity in a neurally defined feature space (based on monkey IT data) predicts human visual search efficiency: targets were more easily identified when subjects were previously adapted to surrounding distractors containing contrastive features represented in neighboring cortical columns. This relates to neural simulations in the contribution of Borji and Itti, suggesting that feature similarity between target and distractors affects whether attention modulates (combinations of) neural gain, shifts in tunings, or sharpening of tunings, to allow for the most informative representations of important stimulus features. Moreover, the employed attentional mechanisms were influenced by task requirements (e.g., object discrimination vs. search), providing a further demonstration of the adaptive nature of feature representations optimized for fast and efficient read-out by higher-level areas. Adding such cognitive top-down influences that warp feature spaces according to salience and relevance, employing vision models with recurrent connections, and defining specific encoding models for each processing stage remains challenging, yet appears necessary for a profound understanding of object representations in the primate brain.

## Concluding remarks

Combining computational and empirical efforts to reveal the neural mechanisms underlying visual object recognition has recently gained momentum. There has been a vast increase in studies employing encoding models to understand how input, transformed to an abstract feature space, predicts measured neural activity. The variety of models under investigation has expanded, ranging from low-level visual descriptors to models that incorporate high-level semantic features. Moreover, advances in high performance computing made it possible to move beyond predefined sets of features, to feature spaces learned from huge and diverse sets of natural world images using deep-learning techniques. Comparing different feature spaces to neural activity can be performed for each measure unit separately (e.g., for each fMRI voxel, see Lescroart et al.) or features can be compared to activation patterns in pre-localized brain regions using similarity estimates (e.g., Aminoff et al.). Recently, Khaligh-Razavi et al. (2014) showed that integrating both approaches, by reweighting and remixing model features via voxel-wise modeling, can lead to higher similarity between models and neural responses in object-selective visual cortex. Direct integration by projecting (population receptive field) voxel models and measured fMRI data in the same brain space might further facilitate comparisons by enabling the use of identical data analysis and visualization techniques for both modeled and measured data (Peters et al., 2012).

The advent of ultra-high field fMRI imaging, large-scale electrocorticographic grids, and dense electrode arrays will provide increasingly rich datasets to study neural activity-patterns with unprecedented detail, yet with sufficient coverage to track reformatting of feature representations from low- to mid- to high-level areas along the VVS. By capitalizing on these increasing opportunities to integrate advanced computer-vision models and large-scale, high-resolution neural datasets, future research can rely on an ever-expanding data mining toolbox to probe neural feature and object representations to uncover the underlying neural “vocabularies.”

## Author contributions

JP wrote the paper with assistance and approval from HO and RG.

### Conflict of interest statement

The authors declare that the research was conducted in the absence of any commercial or financial relationships that could be construed as a potential conflict of interest.
